# Bone metastases from a 1p/19q codeleted and *IDH1*-mutant anaplastic oligodendroglioma: a case report

**DOI:** 10.1186/s13256-019-2061-4

**Published:** 2019-06-28

**Authors:** Mickaël Burgy, Marie-Pierre Chenard, Georges Noël, Khalil Bourahla, Roland Schott

**Affiliations:** 1 Medical Oncology Department, Centre Paul Strauss, 3 Rue de la Porte de l’Hôpital, 67000 Strasbourg, France; 2 Université de Strasbourg, LBP, CNRS UMR 7213, Illkirch, France; 3 Department of Pathology, Strasbourg University Hospital, Strasbourg, France; 4 Radiotherapy Department, Centre Paul-Strauss, Strasbourg, France; 5 Nuclear Medicine Department, Centre Paul-Strauss, Strasbourg, France

**Keywords:** Metastatic oligodendroglioma, Bone metastases, Anaplastic oligodendroglioma

## Abstract

**Background:**

Oligodendroglioma is a rare type of primary brain tumor which, like other malignant gliomas, metastasizes very rarely even when in high-grade form.

**Case report:**

A 36-year-old white man diagnosed 29 months previously as having 1p/19q codeleted anaplastic oligodendroglioma presented bilateral cruralgia and lower limb motor deficits. A computed tomography scan showed multiple osteoblastic bone lesions. The presence of oligodendroglial cells was revealed by bone marrow biopsy and confirmed by immunohistochemical analyses. A positon emission tomography-computed tomography scan confirmed the exclusive involvement of bones.

**Conclusion:**

This case joins less than 20 other reported cases of oligodendroglioma bone marrow metastasis, and is one of only a handful of cases of diffuse bone metastases beyond the axial skeleton. To the best of our knowledge, the early relapse of 1p/19q codeleted anaplastic oligodendroglioma with this distribution of metastases has never been described in the literature.

## Background

It has generally been thought that primary brain tumors rarely metastasize, and that these rare cases only occur inside the central nervous system (CNS). However, a rapid review of the literature demonstrates that this is not true. In a reported series of 116 extracranial metastasis cases, approximately 40% were glioblastomas, 27% medulloblastomas, 16% ependymomas, 10% astrocytomas, and 5% oligodendrogliomas [1]. The oligodendroglioma is a rare neoplasm in adults, and accounts for less than 5% of all primary brain tumors and 5–20% of all glial neoplasms [2]. It can be low grade (grade II) or high grade (grade III, or anaplastic). The prognosis is better than for glioblastoma, with a protracted natural history and a greater response to therapy [3]. The genetic loss of 1p/19q chromosomes corresponds to a distinct tumor entity in oligodendroglioma, which is characterized by a prolonged natural history irrespective of treatment and by a high sensitivity to both radiotherapy (RT) and chemotherapy [3, 4]. As shown in the aforementioned reported series, metastatic relapse is much rarer in oligodendrogliomas than for other malignant gliomas, with fewer than 50 cases reported in the literature [5–42]. Bone seems to be one of the most frequent metastatic sites outside the CNS in this type of glioma, with approximately 20 instances reported since 1970, compared to sites in the lymph nodes, liver, lungs, spleen, or pancreas [12, 14, 29, 31–42]. However, to the best of our knowledge, few cases of patients with diffuse bony metastases extending beyond the axial skeleton have been described to date [34, 36–41].

We report a case of bone metastases from a 1p/19q codeleted and *IDH1*-mutant anaplastic oligodendroglioma 29 months after first diagnosis of a brain tumor.

## Case presentation

A 36-year-old white man presented in April 2017 with a 2-week history of bilateral cruralgia. Following a diagnosis in December 2014 of a right temporoparietal grade III oligodendroglioma with *IDH1* mutation and 1p/19q codeletion, he underwent emergency surgical cerebral decompression for a comatose state secondary to brain herniation, with incomplete resection due to massive cerebral edema. A second surgical resection 1 month later remained incomplete, with residual in-depth disease. He was treated with cranial RT with concomitant temozolomide chemotherapy. Identical chemotherapy treatment was continued from March to December 2015 (standard protocol for high-grade gliomas), receiving six series of treatment that ended 11 months after the second surgery [43]. In January 2016, a cranioplasty was carried out to treat infected craniotomy bone flaps. He was monitored for the following 9 months with regular MRI scans. In August 2016, that is 20 months after surgical resection, a local tumor recurred and was treated with a third subtotal resection. Second-line procarbazine, lomustine, and vincristine (PCV) chemotherapy was initiated following surgery, 4 months before the current presentation.

**Fig. 1:**
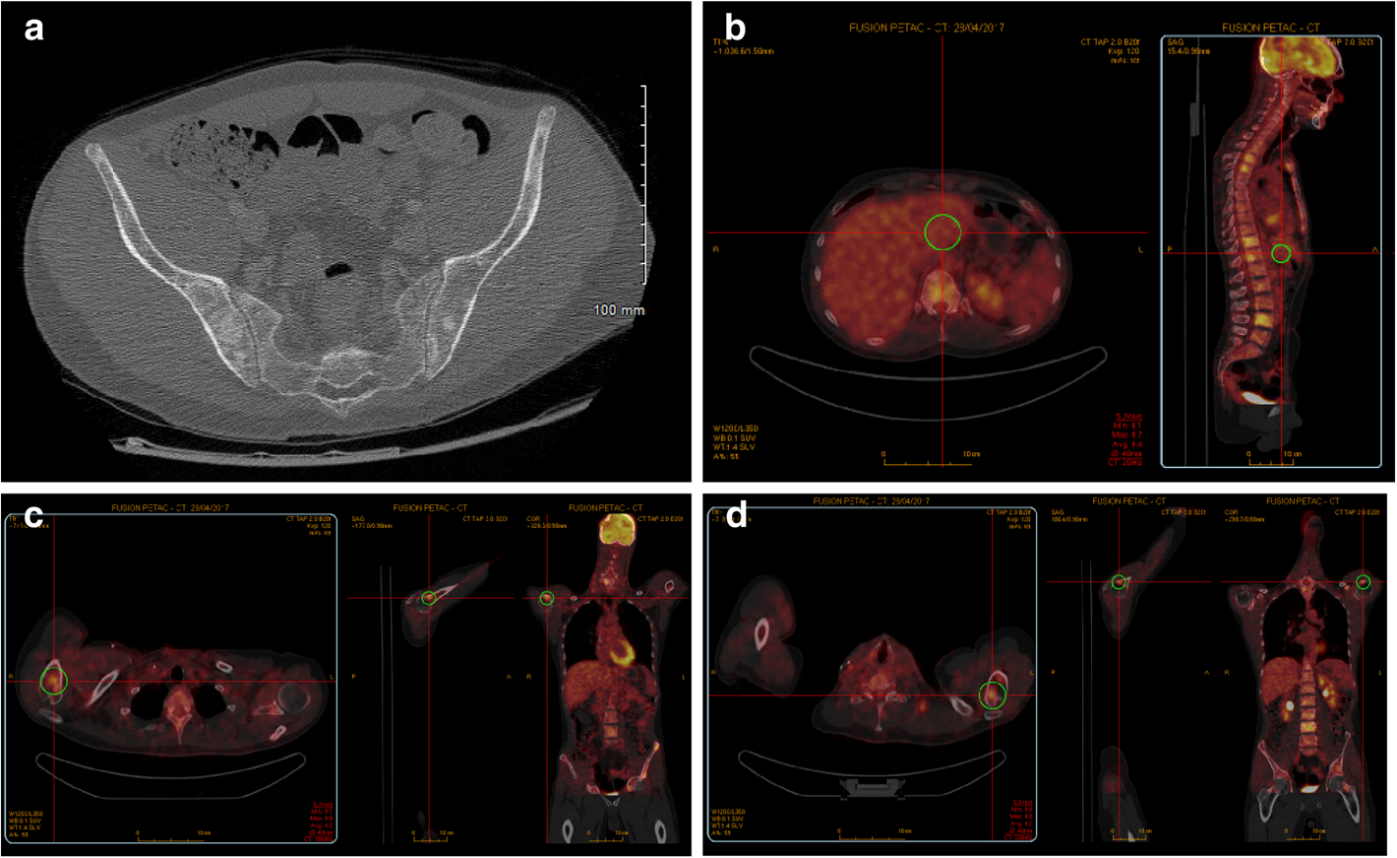
A computed tomography scan of the pelvis shows multiple osteoblastic bone lesions (**a**). Positon emission tomography-computed tomography scan demonstrates the hypermetabolic nature of these spinal bone lesions (**b**) located beyond the axial skeleton, shown here on the right scapula (**c**) and the left humerus (**d**)

**Fig. 2:**
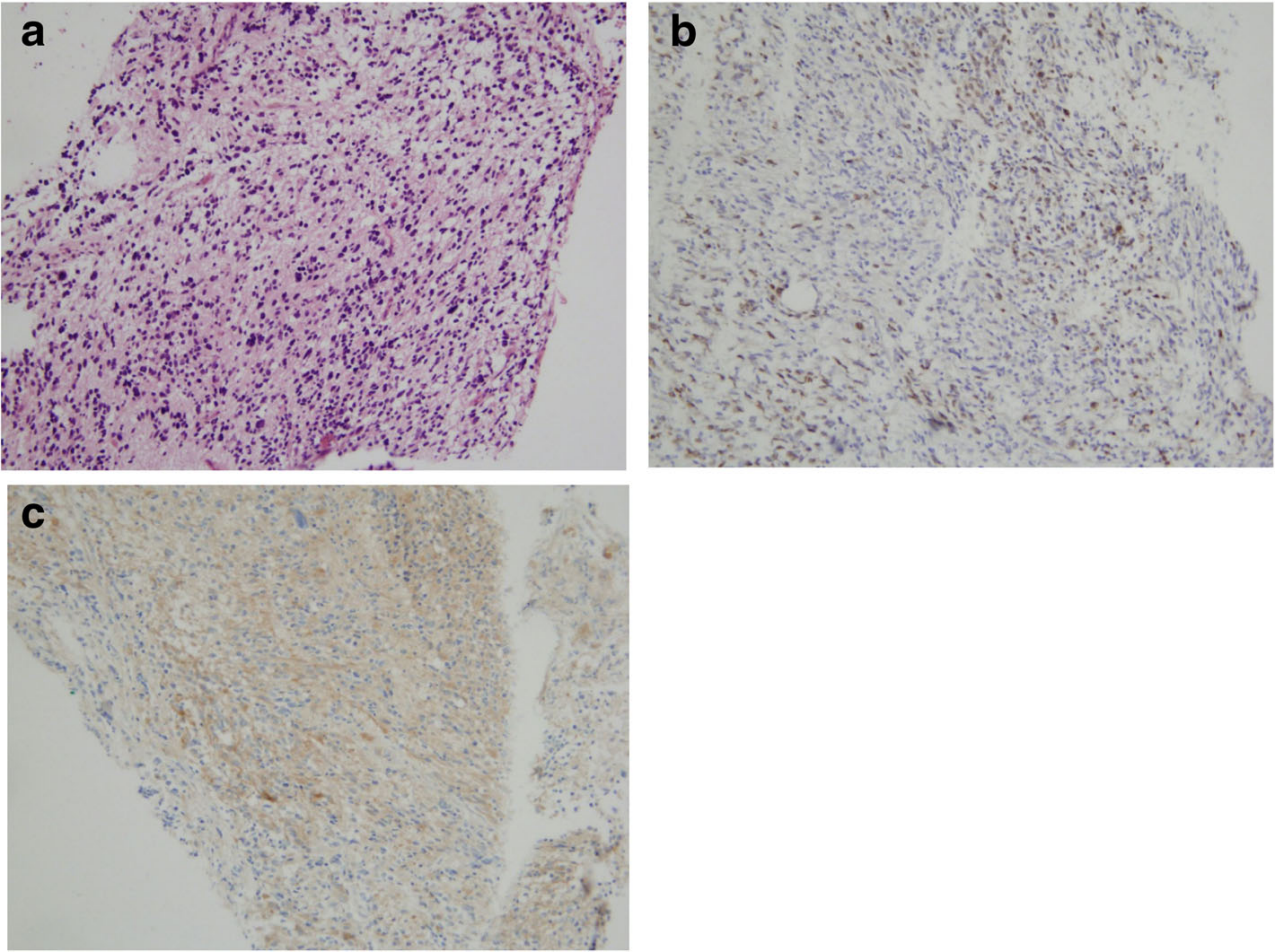
Photomicrographs (magnification × 20) of a hematoxylin and eosin-stained section of metastatic oligodendroglial cells in the bone specimen (**a**), oval tumor cells with pale cytoplasm and irregular cell nuclei with positive Olig2 immunostaining (**b**), and the mutated form (R132H) of *IDH1* (**c**)

A physical examination revealed motor deficits of the lower limbs in addition to pre-existing left-sided hemiparesis and a swollen left supraclavicular lymph node. A computed tomography (CT) scan showed multiple osteoblastic bone lesions scattered throughout his spine, his pelvis and to a lesser extent his ribs, but no lymph adenopathy was identified (Fig. 1a). A positon emission tomography (PET)-CT scan confirmed the presence of the lesions identified in the CT scan and revealed further bone lesions in his pelvis (the right ischium, the pubic area, the left acetabulum, the left part of the sacrum, and the right and left iliac wing), in his sternum with a maximum standardized uptake value (SUVmax) of 4.8, in his right (SUVmax = 5.2) and left humerus (SUVmax = 4.3), and in his right scapula (SUVmax = 4.8). No soft tissue lesions (Virchow’s node included) were found, confirming the exclusive involvement of bones (Fig. 1). The analysis of lymph acquired through fine-needle aspiration of his left supraclavicular lymph node excluded lymph node metastasis. A brain MRI showed local disease progression with an increase in the volume of the right temporoparietal tumor, which had spread to the left temporal region and the superior sagittal sinus. Cytological and biochemical analysis of cerebrospinal fluid excluded carcinomatosis meningitis. Immunohistochemical analyses were carried out on a bone marrow sample from his left iliac crest. A tumoral proliferation of ovoid cells was observed in the medullary cavity. Cells were hyperchromic, had pale cytoplasm and irregular nuclei, and mitosis was occurring: this morphological aspect perfectly reflected the diagnostic for the initial anaplastic oligodendroglioma brain tumor. Immunostaining was positive for glial fibrillary acidic protein (GFAP) and the mutated form of *IDH1*, and therefore excluded any diagnosis other than oligodendroglioma metastasis (Fig. 2). The second-line PCV chemotherapy, initiated following the third subtotal resection 4 months before the current presentation, was continued with two additional cycles in the absence of a real therapeutic alternative and at our patient’s request. It was stopped due to the worsening of his clinical status with positional vertigo, nausea, vomiting, and headache compatible with carcinomatous meningitis, followed by status epilepticus and lethargy; he died after 4 months after the diagnosis of metastases.

## Discussion

Oligodendrogliomas are rare tumors, and represent 5 to 20% of gliomas. Like other glioma tumors, their evolution is characterized by local relapses in the vast majority of cases, and the occurrence of cases of metastasis at sites located outside the CNS is extremely rare. Among these tumors, oligodendrogliomas present the lowest risk of extracerebral metastasis [1]. Different hypotheses have been proposed to explain this phenomenon, which could be due to the anatomical properties of the CNS (absence of a lymphatic system, presence of a neuromeningeal barrier or an environment that is not hostile enough to allow the selection of clones that can metastasize outside the CNS) or could also be explained by a patient history in which death occurred before metastases could spread [1, 44]. However, several publications described how malignant gliomas could bypass the possible aforementioned mechanisms to metastasize outside the CNS. Many factors associated with metastatic relapse outside the CNS have been described, such as repeated intracranial surgery, the presence of a shunt, or prolonged survival [1, 35, 39, 44, 45]. The four intracranial surgeries could explain the metastatic evolution described in this case study. Although there is currently no published evidence, repeated craniotomies may give tumor cells access to the cerebral parenchyma venous pathway, the dura mater, or the scalp, thus bypassing the blood–brain barrier.

Another mechanism that is described is the direct impact of the tumor on the meninges, which increases the permeability of the blood–brain barrier and could therefore facilitate the hematogenous dissemination of tumor cells [31]. Finally, bone flap infection, as observed in this case, could also provide a means of metastatic spread that has not been reported in the literature to date. Here, tumor cells may access vascular pathways following local inflammation, during which vasoactive factors could lead to vasodilation and increased permeability, causing vasogenic edema. However, like the aforementioned mechanisms, the validity of this theory cannot be verified. The delayed occurrence of this metastatic spread may result from a selection of clones following multimodal treatment (RT and chemotherapy), which may make them more aggressive [37, 46]. Tumor cells may also be diffused via the cephalo-rachidian liquid, where they were present in 14% of patients in a series from the 1980s [46]. Our patient presented an oligodendroglioma with 1p/19q codeletion which corresponded to a specific tumor. This molecular change is observed in 30 to 65% of anaplastic oligodendrogliomas [47–49]. These tumors are characterized by a high sensitivity to RT and chemotherapy, and longer survival with and without treatment. The mutation of *IDH1* or *IDH2* genes is observed in all anaplastic oligodendrogliomas with 1p/19q codeletion, and this mutation concerns the *IDH1* gene in the present case study. Only five other cases of metastasis outside the CNS in oligodendroglioma with 1p/19q codeletion have been described in the literature, and no correlation was found between this chromosomal signature and the metastatic relapse [9, 10, 39–41]. The 10-year prolonged survival of patients with this tumor sub-type, compared to 2 years for tumors without this molecular alteration, has been highlighted by previous publications as a possible explanation for the metastatic evolution observed in one of the rare cases reported, in which a 15-year interval occurred between the baseline diagnosis and the metastatic relapse. [40, 50]. Our case does not confirm this hypothesis because metastatic relapse occurred after a particularly short period of 16 months. This case is also singular in terms of metastatic distribution: whereas bone locations with medullary sites appear to be the most frequently reported metastatic sites for oligodendrogliomas (less than 20 cases reported in the literature), only a few descriptions of similar disseminated bone metastases have been reported in the rachis and beyond the axial skeleton [36, 37, 41].

A last explanation could be found in the genetic alteration. Some studies have shown that gliomas result from the accumulation of several distinct chromosomal alterations such as loss of heterozygosity (LOH) on the short arm of chromosome 1 (1p), the long arm of chromosome 19 (19q), and the long arm of chromosome 10 (10q). These acquired genomic alterations in tumor cells have an important role in formulating the prognosis of patients with gliomas, in addition to the histological classification, because some correlate with the clinical outcome. Recently, a correlation between the loss of 10q and anaplastic glial tumor and overall survival was demonstrated [51]. Of note, 10q is the site of *PTEN* encoded on position 10q23 [52]. Today, *PTEN* is considered a tumor suppressor and metabolic regulator [53]. A whole genome analysis on prostate cancer-derived cells showed that *PTEN* loss was robustly associated with the bone metastasis-derived PC3 prostate cancer cell lines but not with the lymph node metastasis-derived LNCaP prostate cancer cell lines [54]. Furthermore, *PTEN* loss was the worst prognostic factor of gastric cancer that developed bone metastasis and was a factor of bone tumor invasion and tumor cell spread in bone [55, 56].

## Conclusion

Metastases from oligodendrogliomas are extremely rare. The novelty of this case lies in the early occurrence of metastatic evolution in a sub-type of oligodendroglioma that habitually has a good prognosis, the singular distribution of metastases, and the different mechanisms that may play a role in tumor cell diffusion.
